# Multiscale Modeling and Data Integration in the Virtual Physiological Rat Project

**DOI:** 10.1007/s10439-012-0611-7

**Published:** 2012-07-18

**Authors:** Daniel A. Beard, Maxwell L. Neal, Nazanin Tabesh-Saleki, Christopher T. Thompson, James B. Bassingtwaighte, Mary Shimoyama, Brian E. Carlson

**Affiliations:** 1 Biotechnology and Bioengineering Center and Center for Computational Medicine, Medical College of Wisconsin, Milwaukee, WI USA; 2 Department of Physiology, Medical College of Wisconsin, Milwaukee, WI USA; 3 Department of Medical Education and Biomedical Informatics and Department of Pathology, University of Washington, Seattle, WA USA; 4 Program in Medical Informatics, University of Wisconsin-Milwaukee, Milwaukee, WI USA; 5 Human and Molecular Genetics Center, Medical College of Wisconsin, Milwaukee, WI USA; 6 Department of Bioengineering, University of Washington, Seattle, WA USA; 7 Department of Surgery, Medical College of Wisconsin, Milwaukee, WI USA

**Keywords:** Semantic annotation, Model merging, Model repositories, Biomedical ontologies, Data dissemination, Model sharing, Mechanistic physiological models, Virtual Physiological Rat

## Abstract

It has become increasingly evident that the descriptions of many complex diseases are only possible by taking into account multiple influences at different physiological scales. To do this with computational models often requires the integration of several models that have overlapping scales (genes to molecules, molecules to cells, cells to tissues). The Virtual Physiological Rat (VPR) Project, a National Institute of General Medical Sciences (NIGMS) funded National Center of Systems Biology, is tasked with mechanistically describing several complex diseases and is therefore identifying methods to facilitate the process of model integration across physiological scales. In addition, the VPR has a considerable experimental component and the resultant data must be integrated into these composite multiscale models and made available to the research community. A perspective of the current state of the art in model integration and sharing along with archiving of experimental data will be presented here in the context of multiscale physiological models. It was found that current ontological, model and data repository resources and integrative software tools are sufficient to create composite models from separate existing models and the example composite model developed here exhibits emergent behavior not predicted by the separate models.

## Introduction

Rather than the result of a single mechanism operating at a single physiological scale, the phenotypes that define a complex disease and/or normal physiological function are often emergent properties of the interaction of a multitude of mechanisms acting (and interacting) across multiple scales. Thus, as the biomedical research community increasingly adopts the view that computational modeling is an essential tool to probe the function of complex nonlinear phenomena, appropriate methods for multiscale simulation will become increasingly important. Blood pressure, for example, is regulated through the interaction of multiple organs and organ systems (neural, cardiac, renal, and endocrine). The multiscale nature of these interacting systems is apparent in neural pathways that modulate the function of the heart on the time scale of the heart beat and the kidney on time scales of minutes to days, influencing whole-organ renal and cardiac function through molecular mechanisms operating on sub-cellular scales. A multiscale synthesis of this knowledge is important because, to continue this example, although a half-century of research on hypertension has identified mechanistic detail at the genetic, cellular, tissue, organ, and system levels, there is no theory of primary essential hypertension that explains its etiology. Since a host of computational models have been developed to simulate the experimentally observed function at each of the individual scales, one potentially useful approach is to determine if and how physiological and pathophysiological function emerges from the integrated operation of models of the component systems.

Such efforts are nontrivial because manual model integration is highly time-consuming, prone to errors in realizing and reproducing models, and requires physiological as well as computational domain expertise. To partially automate this process, we have proposed a practical workflow that makes use of tools for representing and annotating models using unambiguous standards both for instantiating models and for assigning physiological meaning to model components. In addition to piloting this workflow for an example case, we explored how supporting data used for model identification and comparison can be archived in existing repositories in a way that facilitates a transparent and unambiguous connection between models and data sets. The overall goal is to present a perspective of the state-of-the-art in resources for disseminating and using computational models and data in the multiscale physiology arena. In carrying out this exercise it became apparent that, while there is no single standard language to convey physiological meaning or to assign standardized meaning to mathematical modeling components, it is possible to knit together a viable workflow to perform this task using a subset of existing software, standards, and databases. We found that a central tool in this process is SemGen, a software package that helps automate model annotation, composition and decomposition. SemGen leverages the semantic expressivity of the Web Ontology Language (OWL) and the mathematical generality of JSim’s Mathematical Modeling Language (MML) to generate semantically interoperable SemSim^15^,^28^,^29^ models from existing code. These models unambiguously declare the physiological processes they simulate, along with the mathematical representations of those processes. SemGen and JSim are both freely available software packages. SemGen is developed by the Semantics of Biological Processes group at the University of Washington and is available on their website (SemGen, Table 1). JSim, a simulation and modeling analysis software suite developed as part of the Physiome Project also at University of Washington and can be downloaded from their website (JSim, Table 1). This pilot study further reveals a number of gaps in the way existing tools operate and interact that represent major opportunities for current and future development.

**Table 1:** Website links referred to in text

Description	URL
SemGen	http://sbp.bhi.washington.edu/projects/semgen
JSim	http://www.physiome.org/jsim/
VPR Project	www.virtualrat.org
National Centers for System Biology	http://www.systemscenters.org/
BioModels Database	http://www.ebi.ac.uk/biomodels-main/
CellML Project	http://www.cellml.org/
Physiome Model Repository	http://www.physiome.org/Models/
SemanticSBML	http://semanticsbml.org/
Antimony	http://antimony.sourceforge.net/
SABIO-RK	http://sabio.villa-bosch.de/
Saint	http://sourceforge.net/apps/mediawiki/saint-annotate/
Simulation Experiment Description ML	http://sedml.org/
Systems Biology Results ML	http://www.comp-sys-bio.org/tiki-index.php?page=SBRML
Numerical ML	http://code.google.com/p/numl/
FMA	http://sig.biostr.washington.edu/projects/fm/
OPB	http://sbp.bhi.washington.edu/projects/the-ontology-of-physics-for-biology-opb
EBI	http://www.ebi.ac.uk/
IMAG Data Sharing Working Group	http://www.imagwiki.nibib.nih.gov/mediawiki/index.php?title=Data_Sharing_Working_Group
PhysioNet	http://www.physionet.org/
VPR Model 1002	http://www.virtualrat.org/VPR1002/
OpenCell	http://www.cellml.org/tools/opencell

## Virtual Physiological Rat Project

The Virtual Physiological Rat (VPR) project is a current research effort in need of more advanced model integration, model sharing and data sharing (VPR Project, Table 1) and is representative of similar efforts utilizing multiscale computational models to describe complex biological processes and systems. The VPR is supported through an NIGMS National Center for Systems Biology (National Centers for System Biology, Table 1) grant to: (1) develop tools to simulate the integrated cardiovascular function of the rat; (2) identify and validate computer models that account for genetic variation across rat strains and physiological responses to environment (i.e., diet); and (3) use the developed models to predict the physiological characteristics of not-yet realized genetic combinations, derive those combinations, and then test the predictions. Large-scale studies of the influence of genetic variation on cardiovascular phenotypes using standard statistical models reveal that identified genetic determinants of complex diseases can account for no more than a small fraction of the total phenotype variation.^3^,^13^,^22^,^26^ On the other hand, studies of complex traits in chromosome substitution (consomic) mouse and rat strains have shown that “overall phenotypic difference between the parental [strains is] much less than the sum of the phenotypic differences attributable to individual substitutions”.^32^ Resolving these findings (weak association with small additive effects from multiple loci in genome-wide association studies and strong super-additivity apparent in chromosome substitution studies) will require developing a new and sophisticated understanding of the link between genomics and physiology. In short, the VPR project is charged with the grand challenge of transforming our understanding of genotype-phenotype relationships in cardiovascular physiology and disease by synthesizing interactions between many genes, environmental factors, and physiological systems.

To make progress towards this challenge the VPR project is specifically focusing on model development and identification related to cardiovascular system dynamics; whole-body solute transport and energy metabolism; cardiac mechanics, electrophysiology, and metabolism; renal blood flow and solute transport; and statistical methods for mapping genetic variability to variability in model parameters and integrated system function. The goal is to account for integrative function at the subcellular to whole-body levels. For example, the cardiac project involves simulating subcellular biochemical processes, ion handling, excitation–contraction coupling, propagation of electrical signal in the myocardium, and whole-organ mechanics. These processes act on time and space scales that span several orders of magnitude.

One of the major technical challenges facing the VPR project is the task of assembling integrative models from appropriate component modules. Combining computational models of interacting physiological processes in a correct and meaningful way involves a synthesis and transformation of all variables, parameters, and boundary conditions in all components into a new integrated model. Variables invoked in one model may be invoked as parameters or boundary conditions in another. For example, in the relatively simple cardiovascular system example described below, heart rate, which is a model variable in the Bugenhagen *et al*.^5^ model of the baroreflex system, is treated as a fixed parameter in the Smith *et al*.^34^ model of circulatory mechanics. Effective progress of the VPR project, as well as related multiscale integrative physiology research programs, will hinge on reliable archiving and annotating of computational models and relevant data and automated technology for assembling models and associating models with available data.

## Current Standards for Model and Data Dissemination and Integration

### Model Representation Standards

As systems-level modeling has increased in complexity over the years, researchers have recognized the need for representation standards that enable broad model sharing and reuse. The Systems Biology Markup Language (SBML)^20^ and CellML^25^ are two such standards that have emerged over the past decade. Both are XML-based, and this declarative format allows the model specification to exist apart from the code-level implementation. Researchers can therefore process these models in customized ways, but leave the original specification intact. Using XML also provides a structure for capturing metadata about a model, such as its provenance in the literature, curatorial information, and the biological processes it simulates. The SBML, CellML and JSim communities have created online repositories (BioModels Database, CellML Project and Physiome Model Repository, Table 1, respectively) to make models in these formats publicly available, and all currently contain hundreds of curated models.

While both SBML and CellML standards can represent the mathematics of algebraic and ordinary differential equation models regardless of biological scale, in the context of the VPR they have limited capabilities for representing the biological meaning of model contents. SBML’s intended focus is on representing the molecular aspects of the biological processes in a model, and the CellML standard does not yet include a metadata specification for annotating models with biological semantics. For these reasons, the VPR is utilizing modeling languages with metadata frameworks that provide for biological annotation across the molecular, cellular, multi-cellular, tissue, organ, organ systems and population levels. Such frameworks are crucial for a project like the VPR, where we plan to integrate complex models across several biological scales. Additionally the VPR will utilize computational models employing a limited class of partial differential equations used in physiological modeling (e.g., 1-D reaction-convection), which currently can only be represented in an XML based format by the Extensible Mathematical Modeling Language (XMML) used in JSim. SBML and CellML standards have not been extended to represent PDEs however this functionality is planned in the future for both standards.

All three communities (SBML, CellML and JSim) have put forth considerable effort towards standardizing model annotation methods in order to facilitate model merging, decomposition and sharing. A complete, machine-readable description of the biological processes in a model helps automate the cumbersome task associated with model-to-model integration and decomposition into sub-models. For example, the semanticSBML suite of tools (semanticSBML, Table 1) leverages the machine-readable reaction-level descriptions of SBML models to automate the merging of multiple models.^23^ Other model exchange and merging systems have been developed such as Antimony,^35^ System for the Analysis of Biochemical Pathways-Reaction Kinetics (SABIO-RK)^37^ and Saint^24^ which focus on SBML models or a separate reaction kinetics database in the case of SABIO-RK (Antimony, SABIO-RK and Saint, Table 1, respectively). Although this level of model interoperability is available to biochemical reaction modelers, it is not currently available to researchers who model processes at the multicellular, tissue, organ or physiological systems level. To address this limitation, researchers have recently developed the SemSim modeling format, a scalable, semantics-based approach to representing biosimulation models. SemSim leverages the knowledge in publicly available biomedical reference ontologies to thoroughly describe the biophysical meaning of a model’s contents in a machine-readable format. SemSim models contain rich, declarative semantics, implemented in OWL, and because there now exists a set of reference ontologies that describe physical entities and processes across biological scales and research domains, this format provides a more general solution for the automation of modular modeling tasks such as merging and decomposition.

Other modeling standards have been developed utilizing the markup language construct that focus on specifying the use of these models after development. The Simulation Experiment Description Markup Language (SED-ML, Table 1) has been developed to aid in the reproducibility of a simulation experiment across different simulation environments.^36^ Additionally, Systems Biology Results Markup Language (SBRML, Table 1) is designed to associate simulation results with experimental data.^11^ Efforts have been made to facilitate the exchanging and archiving of numerical results through the use of the Numerical Markup Language (NuML, Table 1). All of these standards will be important as developed multiscale models are validated using multiple experimental datasets at multiple scales, however the current focus is to provide model identification and sharing workflows that will support the reuse and integration of models and association with new types of experimental data.

### Use of Ontologies

The purpose of the many biomedical reference ontologies is to define physiological, biological, chemical and physics-based entities and processes in a structured manner. Ontologies are hierarchically structured vocabularies of terms and relationships that are clearly defined and designed to represent and communicate information about a particular scientific domain. For the practice of multiscale physiological modeling, standardizing biological information with organized vocabularies and ontologies^2^,^10^ has proven to be valuable in formally defining components of models and representations of complex systems. Using ontologies allows unambiguous, systematic descriptions of biological entities, processes, and their interrelations.^10^ For example, Gene Ontology (GO) describes gene function through properties of proteins and includes hierarchical information in the three domains of cellular location, molecular functions, and biological processes.^1^,^19^ Key elements in annotating multiscale physiological models with ontology terms include: associating codewords in the model with appropriate unambiguous identifiers; specifying components and subcomponents in the model utilizing the hierarchical structure within an ontology; and linking the model and its components and subcomponents to supporting measured experimental data. Describing multiscale processes in mouse development mathematical models using a combination of GO and Cell Type Ontology (CL) terms has been shown to be extremely effective to provide clear definitions of function and to allow comparison of function under different conditions. This approach shows the potential application of biological ontologies to describe complex processes and systems.^1^ Additionally, the use of multiple ontologies for defining components and subcomponents of multiscale physiological system models will allow them to be compared and integrated to form composite models in an automated manner.

Two other ontologies that are valuable for identifying multiscale models and are widely used are the Foundational Model of Anatomy (FMA) Ontology and the Ontology of Physics for Biology (OPB). The FMA^31^ provides a hierarchical, structured knowledge base of human anatomy that can be investigated in a human readable form but can be interpretable by machine-based applications. The Foundational Model Explorer (FME) web-browsing tool facilitates navigation through the FMA Ontology. The FMA is continuously evolving and is supported by a multidisciplinary group at the University of Washington (FMA, Table 1). The OPB^8^ has also been developed at the University of Washington and provides a rich structure of biophysical properties and processes in an ontological framework (OPB, Table 1). Thus, the OPB is a critical resource for adding semantic detail to physiological models and experimental data. As part of the SemSim framework, the FMA and OPB have been used together to establish composite annotation terms for computational model components that cannot be defined using any single ontology term.^9^ These two ontologies along with GO are the main ontologies used while performing annotation and merging tasks to develop the multiscale computational models described here.

Another important ontology for the systems biology community is the Systems Biology Ontology (SBO) that contains seven sets of orthogonal identifiers used to specify the physiology and mathematics of a system.^10^ SBO is supported by the European Bioinformatics Institute (EBI, Table 1) and is a consensus ontological framework that has been developed to identify and annotate model components including component types, component roles, physical entities and their associated mathematical expressions. However, the current focus of the SBO is on chemical reaction systems and does not currently provide the broad scope required in identifying multiscale models that is facilitated by the combination of the GO, FMA and OPB.

Ontologies are also currently being used and further developed to attach semantic detail to the simulation methods and the numerical results of simulation models. The ontology of simulation procedures known as the Kinetic Simulation Algorithm Ontology (KiSAO) attaches precise identification of individual simulation methodology steps within the model. The Terminology for the Description of Dynamics (TEDDY) has been developed to describe the form of the simulation results, which then can be used to identify experimental results in the validation step of the model. Both of these ontologies will be valuable future components aimed at facilitating the experimental-simulation iterative process of scientific discovery^10^ used by the VPR Project.

#### Data Management and Dissemination

One of the key annotation elements described above is the linking of pertinent experimental data to the whole model and/or components and subcomponents of the model. Standardization of data formats, structural elements and their attributes facilitates integration of different models and provide structures on which innovative analysis, and presentation tools can be built and experimental and computation model design can be reevaluated. Moreover, formalizing models using guidelines on how to encode information, and standardizing data through the use of ontology terms will enable unambiguous transfer and interpretation of the information and data.^4^,^16^ This issue of data sharing and management is a current working group topic under the Interagency Model and Analysis Group (IMAG), an interagency consortium working on several projects involved with multiscale modeling and dissemination (IMAG Data Sharing Working Group, Table 1).

To this end, the development of feasible dissemination platforms and standardized data-management systems are currently being proposed. Ghosh *et al*.^16^ focuses on two core aspects of management standards besides the use of ontologies, including minimum information and file formats. Minimum information is defined as the least amount of metadata to allow duplication of an experiment. File format standards such as XML define how the minimum information should be stored so that it can be easily processed by a machine.^16^ Such measures allow us to develop and facilitate an in depth understanding of physiological models and make them available to different research communities.

A current data management system is PhysioNet, a resource for complex physiologic signals, time series, images, and relevant open source software (PhysioNet, Table 1). PhysioNet is a multicenter collaboration^17^ funded by the National Institute of Biomedical Imaging and Bioengineering (NIBIB) and the NIGMS. One major component of PhysioNet is PhysioBank, which is an archive of physiologic signals, time series, and related clinical data. “PhysioBank functions as a repository for selected physiologic signals and time series data from published studies in peer-reviewed journals”.^27^ Datasets from PhysioBank can be downloaded in several different formats including MATLAB readable .mat files, text files and an additional format readable by an open source WaveForm DataBase (WFDB) software package.

### Model Integration Using SemGen

Currently one of the most advanced tools for standardizing and integrating models is SemGen, a Java-based experimental application built to create, annotate, decompose, merge and encode models for simulation, which is freely available (SemGen, Table 1). At the heart of the application is the SemSim architecture,^15^,^28^,^29^ a declarative model description format separate from code-level implementations used to capture model semantics in a standardized manner. One of the advantages of SemGen is that SemSim versions can be created from any biological model that compiles within JSim, a freely available, general-purpose simulation environment (JSim, Table 1). This includes most curated SBML and CellML models as well as models coded in JSim’s MML. Once translated into the SemSim format, SemGen provides tools for applying deep semantic annotations to the model elements. This annotation step captures the biological meaning of the simulated processes in the model, and is the key for making SemSim models modular and interoperable. Once a SemSim model is thoroughly annotated, a user can perform model decomposition and integration tasks at the biological level of conceptualization, rather than the code level. For example, using the Extractor tool within SemGen, a user can extract the heart component out of a larger cardiovascular model simply by selecting the heart-related physical entities among the model’s annotations. No manual coding is required to create a compilable heart submodel, however, because this submodel is now separated from the larger system, input values must be specified by the user for the submodel simulation to run. These unspecified inputs are readily identified visually within the SemGen Extractor tool.

When merging two SemSim models, SemGen examines the points of semantic overlap between the models in order to create a biologically consistent interface between them. That is, if two models simulate the same biological property using two different computational formulations, then the user must choose which formulation to preserve in the merged model. This resolution step creates an interface point between the models, coupling them into a merged system. For example, if a user merges a model that simulates left ventricular contraction with a model of the aorta, and both represent blood flow through the aortic valve, SemGen can identify this semantic overlap and prompt the user to create an appropriate interface between the models where aortic valve flow is computed from the left ventricle dynamics and drives flow into the aorta. In the following section we discuss a merging task we performed with SemGen where we combined a cardiovascular system model with a baroreceptor model to create a coupled system that includes baroreceptor feedback control of arterial blood pressure.

## Examples from Current VPR Effort

### Example 1: Cardiovascular Systems Dynamics

**Figure 1:**
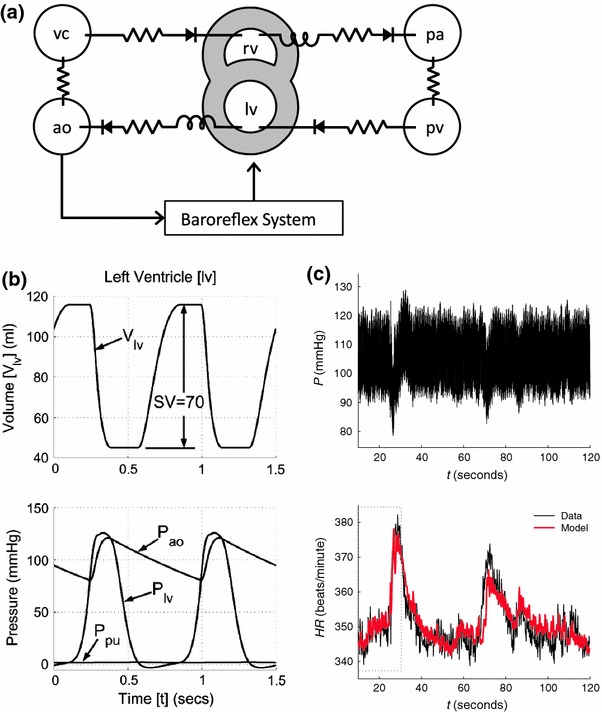
Integrated model of cardiovascular system dynamics. (a) Integrated model as a combination of the simple cardiovascular model of Smith *et al*.^34^ and the baroreflex model of Bugenhagen *et al*.^5^ The two ventricles of the heart are labeled “lv” and “rv,” for left and right ventricle. Two compliant compartments, labeled “ao” and “vc” for aorta and vena cava, represent the systemic circulation. Similarly, two compartments labeled “pa” and “pv” for pulmonary artery and pulmonary vein represent the pulmonary circulation. (b) Example output from the Smith *et al*. model, reproduced with permission. (c) Example fit of simulated to measured heart rate for the baroreflex model of Bugenhagen *et al*., reproduced with permission

In a pilot project relevant to the VPR project, we assembled a composite cardiovascular dynamics model by combining the cardiovascular dynamics model of Smith *et al*.^34^ and the baroreflex systems model of Bugenhagen *et al*.^5^ The integrated composite model is diagrammed in Fig. 1a. The published parameterization of the Smith *et al*. model represents human cardiovascular dynamics, as illustrated in Fig. 1b; the Bugenhagen *et al*. model is parameterized based on data from rat, as illustrated in Fig. 1c.

The composite model was built by annotating the Smith *et al*. and Bugenhagen *et al*. models, primarily using the FMA,^31^ Gene Ontology^2^,^19^ and Ontology for Physics in Biology,^8^ and combining the models using SemGen. The Smith *et al*. model parameter values were adjusted to represent rat cardiovascular dynamics. The complete workflow of the merging process involved taking CellML versions of the baroreflex system and the CV dynamics model of Smith *et al*. and converting them into MML versions using JSim. These MML versions were then loaded into SemGen and were annotated individually to produce SemSim versions. (Whereas we used a previous SemGen version that requires converting SBML and CellML models to MML before they can be translated into the SemSim format, the latest available version can convert SBML and CellML models directly into SemSim models.) In SemGen, we merged these SemSim versions into the composite CV dynamics model and then encoded the model in MML for simulation in JSim and conversion to CellML. Automatic conversion from MML to CellML for the composite model is now currently supported with JSim version 2.06 and later.

During the merging process two points of semantic equivalency were identified and resolved within SemGen, namely the pressure in the aorta and the heart period, which were represented in both models. However, some additional manual modifications had to be made to generate the version of the model that produced the Valsalva maneuver shown in this example. These changes were: addition of the Valsalva perturbation, adjustment of CV model parameters to reflect rat instead of human physiology, addition of left, right ventricular and septal wall elastance terms that are functions of heart rate, introduction of a more complicated expression for driving heart contraction that is a function of the changing heart period, and conversion of units of kPa in the merged model to mmHg. The identification of most of these changes is not within the current scope of the SemGen merging tool and represent alterations to the basic merged model which was automatically generated; however, the manual conversion of kPa to mmHg could have been avoided if compatible unit systems were used in the development of the CellML versions of the original individual models. The original CellML, MML and annotated SemSim versions of the individual models along with annotated SemSim, MML and MATLAB (for comparison) versions of the final composite model and a detailed description of the merging workflow are available for download on the VPR model page (VPR Model 1002, Table 1). All simulations in this example were run on a desktop computer (MPC, Intel Pentium 4 CPU, 280 GHz, 3 GB RAM).

**Figure 2:**
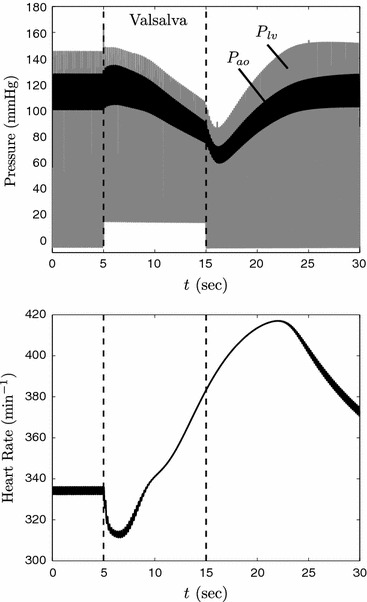
Simulation of integrated cardiovascular mechanics model. The upper panel shows simulated left-ventricular and aortic pressure during baseline conditions, during a transient 20 mmHg increase in thoracic pressure (period labeled “Valsalva,” and during recovery. The lower panel shows predicted heart rate during the simulation

The combined model has the ability to simulate phenomena that cannot be captured by either the Smith *et al*. model or the Bugenhagen *et al*. model on its own. As an example, Fig. 2 plots simulated left-ventricular pressure (*P*_lv_), aortic pressure (*P*_ao_), and heart rate in response to a transient increase in thoracic pressure. A transient increase in thoracic pressure reduces blood flow into the right atrium, resulting in reduced cardiac output and decreased aortic pressure and is commonly referred to as the Valsalva maneuver. This procedure can be performed by simultaneously holding ones breath while contracting the abdominal muscles and is a noninvasive way to perturb cardiac function. Specifically in this simulation, during the interval marked “Valsalva” the thoracic pressure is increased from the baseline value of −4 mmHg to the value of 16 mmHg. After 10 s of elevated pressure, thoracic pressure is returned to the baseline value. Several interesting phenomena emerge from the response to and recovery from this simulated Valsalva maneuver.

During the initial phase of the response, the increase in thoracic pressure increases systemic pressure through direct mechanical influence on the heart and systemic vessels. The increase in aortic pressure causes a transient decrease in heart rate *via* the baroreflex. Following the initial response of increased pressure, pressure begins to drop as a result of elevated thoracic pressure impeding venous flow to the heart. Heart rate increases in response to the reduction in pressure predicted over the second phase of the Valsalva interval.

Both the predicted initial reduction and the overshoot in recovery of heart rate represent emergent phenomena that can be compared to data from subjects performing the Valsalva maneuver. Indeed, measurements in healthy human subjects consistently show an initial decrease and recovery overshoot in heart rate in response to the Valsalva maneuver.^12^

### Example 2: Vascular Blood Flow Regulation

The VPR Project is also currently developing multiscale models used to represent blood flow regulation in the peripheral vasculature. Individual microvessels in the vasculature are known to respond to acute mechanical stimuli by dilating and constricting thereby regulating blood flow through them. Models of subcellular and cellular components inside the vessel wall can be integrated with a model of vessel wall mechanics to describe the vessel diameter response to stimuli acting at the cellular level. At the subcellular level a model of vascular smooth (VSM) muscle force generation by Hai and Murphy^18^ is used to simulate the force generated in a VSM cell at various cytosolic Ca^2+^ levels. At the whole cell level, models of VSM^21^ and vascular endothelial (VE)^33^ electrophysiology and ion transport are incorporated. Integrating these three models together requires additional development of the electrical communication and ion transport between the VSM and VE. Then these models can be integrated with the vessel wall mechanics model^7^ where vessel wall circumferential stress and shear stress on the endothelial cells are used to determine Ca^2+^ entry into the VSM and NO production in the VE respectively.

**Figure 3:**
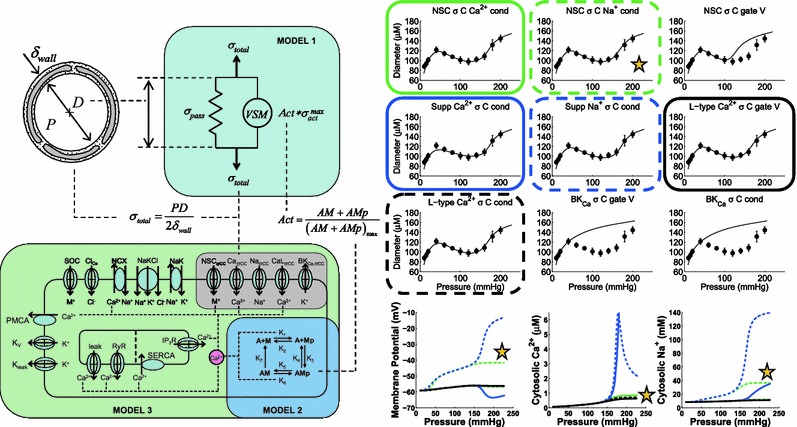
Integrative cellular based vessel model of the steady-state myogenic response. Vessel wall stress controls Ca^2+^ influx, which in turn determines level of VSM contraction and vessel diameter. Nine hypothetical stress-controlled ion channels were independently inserted into the model to show (upper panel of nine figures) six possibilities (boxed plots) to fit experimental data. However known trends of membrane potential, cytosolic Ca^2+^ and cytosolic Na^+^ are only matched by simulations (star) of the stress-controlled Na^+^ influx through non-selective cation (NSC) channel. Adapted from Carlson and Beard^6^ reproduced with permission

The VSM force generation, VSM electrophysiology and ion handling, and vessel wall mechanics models were previously integrated manually^6^ to evaluate which channels in the vascular smooth muscle (VSM) had the ability to describe the vessel diameter response to changes in vessel wall stress induced by increased intraluminal pressure (Fig. 3). While manually integrating these models, several issues arose that could have been identified quickly if a tool such as SemGen had been used when initially developing these separate models. First, unit differences between all the models could have been identified automatically. This is currently automatically done when SemGen merges multiple SemSim model representations. Second, integration with SemGen would have identified that the vessel wall stress from the vessel wall mechanics model was an output variable with no connection to the VSM electrophysiology and ion handling model. Recognition of this unconnected variable identifies the need for some additional model code to be written to represent a feedback loop present in the integrated model but not represented in any of the individual models. Third, only a portion of the VSM electrophysiology and ion handling model was necessary in this composite model. Because SemGen currently provides submodel extraction tools, we could have extracted only the required portion and reduced computational time simulating the integrated system. Submodel extraction becomes a critical function as the multiscale models become larger and optimization to experimental data requiring multiple simulations is performed. Finally, if the individual models were converted into the SemSim exchange format the individual along with the merged models could subsequently be translated into MML and CellML for model sharing and dissemination. The VSM electrophysiology and ion handling model was originally coded in Fortran while the remaining models were coded in MATLAB and JSim. All models had to be recoded into MATLAB in Carlson and Beard^6^ which limited the sharing of this model with the wider community. Translation from SemSim to MML and CellML is currently supported within SemGen and translation to MATLAB is in development.

**Figure 4:**
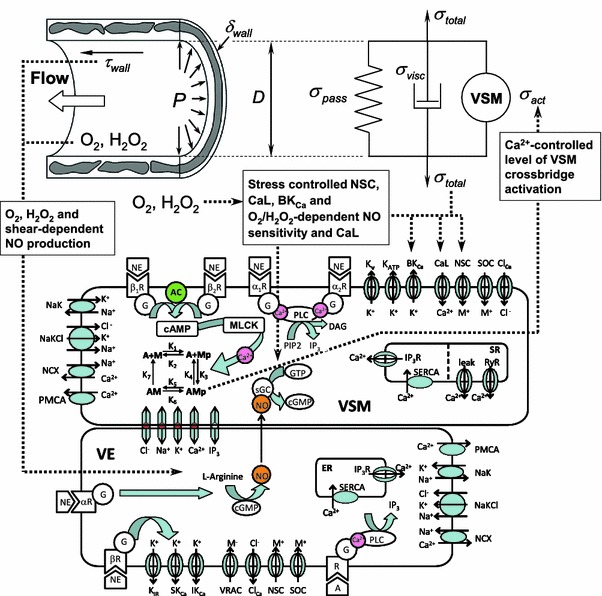
Integrated model of vascular blood flow regulation in a single vessel incorporating response to vessel wall stress induced by changes in intraluminal pressure and response to shear stress on VE cells due to changes in blood flow through the vessel. The VSM cell model incorporates a previous model by Kapela *et al*.^21^ with the addition of α_2_-, β_1_- and β_2_-adrenoceptors and stress-controlled Ca^2+^ influx. The VE cell model utilizes an existing model by Silva *et al*.^33^ adding α- and β-adrenoceptors and O_2_- and H_2_O_2_-dependent NO production. The vessel wall mechanics model is similar to a previous model by Carlson and Secomb^7^ with the addition of a viscous element

Extension of this vascular blood flow regulation model will include further incorporation of VE electrophysiology and ion handling along with addition of viscoelastic components in the vessel wall to model transient instead of steady state behavior. A vision of this model can be seen in Fig. 4. SemGen will be an integral part of the assembly of this multiscale blood flow regulation model.

## State of the Art, Needs and Challenges

The ontologies, modeling standards and software tools—in particular GO, FMA, OPB, CellML, MML, JSim SemSim and SemGen—discussed in this paper form an excellent suite of resources for the merging, extraction and dissemination tasks required for modeling physiological systems. There exists overlap between different ontology standards, and of course across modeling languages and simulation environments. However instead of being considered a disadvantage in forming modeling standards and practices, this overlap could be regarded as a rich environment from which to cultivate an appropriate methodology for model merging and sharing. Ideally there would exist a common platform for merging and dissemination tasks that can bring together various relevant ontologies and multiple modeling languages to produce unambiguously annotated computational models that can be invoked in a variety of simulation environments. Here we developed and applied an annotation and merging workflow based exclusively on CellML, MML and SemSim which was used to create the composite model of Example 1.

**Figure 5:**
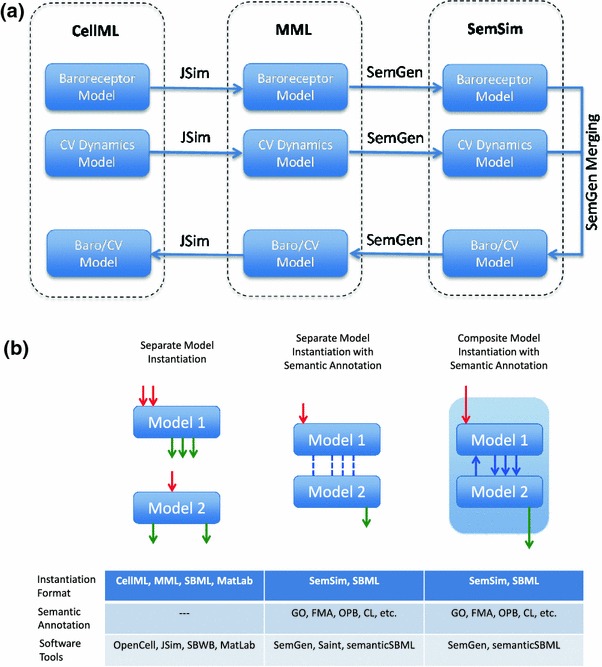
Description of workflow using JSim and SemGen used to develop the cardiovascular system dynamics model (a) and generic workflow necessary for the annotation and merging of computational models (b). In Example 1 CellML and MML were used as model instantiation languages while SemSim was the language used to capture both model instantiation and semantic annotation. JSim was used to facilitate the translation from CellML to MML and annotation, merging and translation of the composite model to MML were performed with SemGen. In (b) red arrows indicate model input, green arrows indicate model output and blue arrows indicate internal connections between Model 1 and Model 2 in the composite model

CellML models were used here since CellML is specifically designed for biological models and its modular design is well suited for describing the interaction of multiple systems. The repository of CellML models currently holds over 500 models and because nearly all CellML models can be translated into the SemSim format, there is great potential for translating, annotating and merging them within SemGen. Translation from CellML to MML is facilitated within JSim, which is a simulation environment that provides an extensive suite of analytical tools such as loops, sensitivity analysis, and parameter optimization for analysis and visualization. All semantic annotation and mathematical descriptions are viewed within the SemGen environment. The workflow presented here along with similar semantic annotation and merging tools available for SBML models are presented in Fig. 5.

The SemSim versions of the models created within SemGen are able to contain the mathematical representation of the model and the semantic meaning of the model as described during the annotation process. Once both of these representations are joined within SemGen the models can be systematically and reproducibly merged into larger multiscale models and/or portions of the model can be extracted for reuse and dissemination. These SemSim models can be translated into MML and CellML for simulation and visualization in JSim and OpenCell (OpenCell, Table 1), respectively, and have the potential to be translated into languages used by other simulation environments such as MATLAB. There are currently other platforms for annotation of SBML such as Saint^24^ and semanticSBML.^23^ However for the multiscale integrated models currently being developed for the VPR, CellML, MML and SemSim are more appropriate exchange formats.

These integrated models, annotated based on formal ontologies, provide a framework for linking genes to function and disease phenotypes. Indeed, one of the goals of many of the ontologies that are being adopted by the VPR is to facilitate the linking of genes to function, independently of the use of computational models. For example, the Gene Ontology is a particularly valuable resource for terms describing high-level integrated physiological processes and complex disease phenotypes. The task of annotating the whole genome for all relevant process is doomed to be forever incomplete. (For example, the hundreds of genes associated with the term “regulation of blood pressure” can represent only small fraction of all the gene products involved in all of the cellular processes that contribute to mechanical pumping of blood, maintenance of vascular tone in various circulatory beds, and nervous and hormonal control mechanisms. Furthermore, it is practically impossible to enumerate all of the high-level processes necessarily associated with a single gene.^30^) Yet, while forever incomplete as an ontology of attributes of genes, the Gene Ontology is an immensely useful knowledge base and source of structured standardized terminology on physiology and disease. By linking model components (including mathematical expressions, individual variables and parameters) to standardized terms, connections between model predictions and scientific literature and other databases can be automated and managed using semantics-based technology. Similarly, associating relevant identifiers with appropriate gene products that are invoked in cell models will streamline the process of exploring links between the low-level processes associated with specific gene products and the high-level function predicted by integrated multiscale models.

One of the major shortcomings of the workflow applied here and illustrated in Fig. 5 is that a portion of the merging process requires modifications to be recognized and implemented manually by the user. These modifications were compounded by the use of inconsistent unit systems between the original versions of two models of Example 1. Some modifications will continue to be a manual process that requires special knowledge to be provided by the user such as alteration of fixed model parameters to reflect the new system represented by the merged model. However many issues are already automated during the model merging process such as the identification of identically named codewords in two or more of the models to be merged or the recognition of model overlap in need of resolution by the user. Often the user immediately modifies the merged model as we did in Example 1 but the automatically generated merged model serves as an excellent starting point for these modifications. Pilot studies such as that presented here will help to identify the strength and weaknesses of these current tools. Thus it is suggested that the workflow of Fig. 5 is simply a snapshot of the current utility of these tools for the merging, reuse and dissemination of multiscale models and as such represents an opportunity for tool development and refinement toward the common objectives of the CellML, JSim and SBML communities.

The use of the current set of model description and annotation tools even in their developmental stages shows a great deal of promise in streamlining the merging, development and testing of multiscale models. The extra effort on the front end of attaching ontological information to these models will facilitate merging into larger multiscale models, extraction of submodels for reuse and sharing, and linking of these multiscale models to relevant experimental data for parameterization and validation. These tools however are not a substitute for detailed knowledge at the physiological level and of related simulation methods. In fact, with these merged multiscale models the method of simulation, determination of initial or boundary conditions, or numerical representation of the experimental protocol of the individual components will not always translate directly to the merged model. This reality has driven the development of KiSAO and TEDDY,^10^ ontologies for the description of simulation methods and results. Similar efforts will prove to be a further aid in the iterative process of simulation and experimentation driving the understanding of mechanistic physiological system interaction described within the VPR project. In cases of multiscale model development where a model is formed by merging two or more existing models, an additional set of user supplied code is often required to complete the merged model.^14^ Tools such as SemGen can help identify these “loose ends” that need attention from the researcher while automatically resolving the variables and parameters that are recognized as equivalent. Recognizing these equivalencies is necessary for guiding users through the merging process; however, recognizing semantic *similarity* will also provide information that is critical to merging tasks (as in recognizing the similarity between heart rate and heart period). The SemGen developers are currently exploring how to apply existing semantic similarity measures for composite annotations in SemSim models.

The exercise of applying our currently available model annotation and integration workflow to the models of Example 1 and projecting them onto the future task of developing models such as that proposed in Example 2 demonstrates the feasibility of the current methodology and its potential as an integral part of the development and dissemination of models within the context of the VPR Project. In the Example 1 merging task we found that the models could be annotated at a resolution sufficient to achieve the merging of the two models into a composite model that possesses emergent properties, which are not present in the individual submodels. When considering multiscale models such as that shown in Example 2 we anticipate that the SemGen platform will facilitate the integration of additional submodels (such as α_2_-, β_1_- and β_2_-adrenoceptors) to be easily integrated into the previously developed VSM model to describe a larger range of cellular function within the multiscale system. In addition, attaching mechanistic mathematical descriptions to terms in the ontologies and experimental data enriches the information associated with these terms and data leading to an integrative understanding of what they represent.
